# Functional Genomics in Chickens: Development of Integrated-Systems Microarrays for Transcriptional Profiling and Discovery of Regulatory Pathways

**DOI:** 10.1002/cfg.402

**Published:** 2004-04

**Authors:** L. A. Cogburn, X. Wang, W. Carre, L. Rejto, S. E. Aggrey, M. J. Duclos, J. Simon, T. E. Porter

**Affiliations:** 1 Department of Animal and Food Sciences University of Delaware Newark DE 19717 USA; 2 Statistics Program Department of Food and Resource Economics University of Delaware Newark DE 19717 USA; 3 Department of Poultry Science University of Georgia Athens GA 30602 USA; 4 Station de Recherches Avicoles Institut National de la Recherche Agronomique Nouzilly 37 380 France; 5 Department of Animal and Avian Sciences University of Maryland College Park MD 20742 USA

## Abstract

The genetic networks that govern the differentiation and growth of major tissues of economic importance in the chicken are largely unknown. Under a functional genomics project, our consortium has generated 30 609 expressed sequence tags (ESTs) and developed several chicken DNA microarrays, which represent the Chicken Metabolic/Somatic (10 K) and Neuroendocrine/Reproductive (8 K) Systems (http://udgenome.ags.udel.edu/cogburn/). One of the major challenges facing functional genomics is the development of mathematical models to reconstruct functional gene networks and regulatory pathways from vast volumes of microarray data. In initial studies with liver-specific microarrays (3.1 K), we have examined gene expression profiles in liver during the peri-hatch transition and during a strong metabolic perturbation—fasting and re-feeding—in divergently selected broiler chickens (fast vs. slow-growth lines). The expression of many genes controlling metabolic pathways is dramatically altered by these perturbations. Our analysis has revealed a large number of clusters of functionally related genes (mainly metabolic enzymes and transcription factors) that control major metabolic pathways. Currently, we are conducting transcriptional profiling studies of multiple tissues during development of two sets of divergently selected broiler chickens (fast vs. slow growing and fat vs. lean lines). Transcriptional profiling across multiple tissues should permit construction of a detailed genetic blueprint that illustrates the developmental events and hierarchy of genes that govern growth and development of chickens. This review will briefly describe the recent acquisition of chicken genomic resources (ESTs and microarrays) and our consortium's efforts to help launch the new era of functional genomics in the chicken.


**Comparative and Functional Genomics**
*Comp Funct Genom* 2004; **5**: 253–261.
Published online in Wiley InterScience (www.interscience.wiley.com). **DOI:** 10.1002/cfg.402

**Conference Review**

### Functional genomics in chickens: development of integrated-systems microarrays for transcriptional profiling and discovery of regulatory pathways

L. A. Cogburn[1]*, X. Wang[1], W. Carre[1], L. Rejto[2], S. E. Aggrey[3], M. J. Duclos[4], J. Simon[4] and T. E. Porter[5]
[1]*Department of Animal and Food Sciences, University of Delaware, Newark, DE 19717, USA*
[2]*Statistics Program, Department of Food and Resource Economics, University of Delaware, Newark, DE 19717, USA*
[3]*Department of Poultry Science, University of Georgia, Athens, GA 30602, USA*
[4]*Station de Recherches Avicoles, Institut National de la Recherche Agronomique, 37 380 Nouzilly, France*
[5]*Department of Animal and Avian Sciences, University of Maryland, College Park, MD 20742, USA*

**Correspondence to:*
*L. A. Cogburn, Department of Animal and Food Sciences, University of Delaware, 45 Townsend Hall, Newark, DE, 19717, USA.*
*E-mail: cogburn@udel.edu*

Received: 16 February 2004
Accepted: 16 February 2004

### Abstract

**The genetic networks that govern the differentiation and growth of major tissues of economic importance in the chicken are largely unknown. Under a functional genomics project, our consortium has generated 30 609 expressed sequence tags (ESTs) and developed several chicken DNA microarrays, which represent the Chicken Metabolic/Somatic (10 K) and Neuroendocrine/Reproductive (8 K) Systems (http://udgenome.ags.udel.edu/cogburn/). One of the major challenges facing functional genomics is the development of mathematical models to reconstruct functional gene networks and regulatory pathways from vast volumes of microarray data. In initial studies with liver-specific microarrays (3.1 K), we have examined gene expression profiles in liver during the peri-hatch transition and during a strong metabolic perturbation — fasting and re-feeding — in divergently selected broiler chickens (fast vs. slow-growth lines). The expression of many genes controlling metabolic pathways is dramatically altered by these perturbations. Our analysis has revealed a large number of clusters of functionally related genes (mainly metabolic enzymes and transcription factors) that control major metabolic pathways. Currently, we are conducting transcriptional profiling studies of multiple tissues during development of two sets of divergently selected broiler chickens (fast vs. slow growing and fat vs. lean lines). Transcriptional profiling across multiple tissues should permit construction of a detailed genetic blueprint that illustrates the developmental events and hierarchy of genes that govern growth and development of chickens. This review will briefly describe the recent acquisition of chicken genomic resources (ESTs and microarrays) and our consortium's efforts to help launch the new era of functional genomics in the chicken. Copyright © 2004 John Wiley & Sons, Ltd.**

### Introduction

The chicken was first domesticated from red jungle fowl (*Gallus gallus*) in south-east Asia (now Thailand) more than 8000 years ago [1]. Domestic chickens (*Gallus domesticus*) were soon found along the Yellow River (Huang He) in north-east China and eventually they were carried into Europe through Persia and by the Roman conquests. The early domestication of the chicken played a significant role in the global spread of a flourishing human culture [2]. Today the domestic chicken continues to serve mankind as a widely-used biological model and an important global source of high-quality protein from meat and eggs. Until recently, the chicken had

Copyright © 2004 John Wiley & Sons, Ltd.

received less attention for comparative and functional genomics, mainly due to a low number of expressed sequence tags (ESTs) and the lack of a completed genome sequence. Currently, there are only 8868 chicken Unigenes in GenBank (**http://www.ncbi.nlm.nih.gov/entrez/query.fcgi?db=unigene**), which is about half the number of Unigenes listed for pigs, or cattle. Over the past 3 years, there has been a remarkable increase in the number of chicken ESTs entered into the dbEST division of GenBank (Table 1); this feat has quickly advanced chicken to the sixth-largest collection, with 460 385 ESTs — first place being held by the human collection of 5 471 545 ESTs (**http://www.ncbi.nlm.nih.gov/dbEST/dbEST_summary.html**). Perhaps more remarkable, a 6.6× coverage sequence of the chicken genome has just been completed by the National Human Genome Research Institute (NHGRI) at the Washington University Genome Sequencing Center (**http://genome.wustl.edu/projects/chicken/**) [3], within the predicted 1 year deadline [4]. In the near future, the availability of these genomic resources should drive the chicken towards the forefront of developmental and systems biology, and promote its use as a model for comparative and functional genomics research [5] (see ChickNET at: **http://www.chicken-genome.org/**). The present review will recount some of these recent acquisitions and, in particular, our consortium's efforts to help launch the new era of functional genomics in the chicken [6].

### Development of genomic resources for chickens

### Making a comprehensive catalogue of genes expressed in chicken tissues

In 2000, only a few thousand chicken-expressed sequence tags (ESTs) were found in GenBank; these ESTs were derived primarily from lymphoid tissue [7,8]. Under a USDA–IFAFS/Animal Genome Program consortium project for functional genomics in chickens, we initiated the first comprehensive EST discovery project in chickens, with high-throughput sequencing of a number of single and multiple-tissue cDNA libraries, which were genetically and developmentally complex [6] (see Acknowledgements). This original EST sequencing effort has been completed with the single-pass 5′-end sequencing of 42 870 chicken cDNA clones (Table 1) from a set of tissue-specific cDNA libraries (**http://www.chickest.udel.edu**)

**Table 1.** Chicken expressed sequence tags (ESTs) in the dbEST division of GenBank

| Institution | Tissue | GenBank entries |
|---|---|---|
| University of Delaware (UD) | Adipose tissue | 6479 |
| | Liver | 5256 |
| | Skeletal muscle/growth plate | 5767 |
| | Pituitary/hypothalamus/pineal | 8719 |
| | Reproductive tract (oviduct, ovary, testes) | 3041 |
| | Lymphoid tissues (activated T cells, spleen, bursa, thymus, activated macrophages) | 18 591 |
| | UD Total | 47 853 |
| BBSRC* | [1]Embryo (9 tissue-specific libraries) | 330 096 |
| | [2]Adult (12 tissue-specific libraries) | |
| Heinrich-Pette-Institute | Bursa of Fabricius; DT40 cells | 23 023 |
| Fred Hutchinson Cancer Research Center (FHCRC) | Bursa of Fabricius | 1116 |
| Roslin Institute | Brain, embryo | 5194 |
| USDA/ARS/BARC | Small intestine infected with coccidia | 14 376 |
| INRA/Agenae | Brain, multi-tissues, ovary, embryos, hypothalamus, skin, pituitary gland; embryo to adult | 23 273 |
| University of Sao Paulo ESALQ | Limb buds (e3.5, e4.5 and e6) | 2132 |
| Total EST sequences | | 460 385 |

* The Biotechnology and Biological Sciences Research Council (BBSRC) Consortium (University of Manchester Institute of Science and Technology, University of Nottingham, University of Dundee and the Roslin Institute).
[1] Embryo: day 16, stages 10, 20, 21, 22 and 36.
[2] Adult: brain, heart, pancreas, adipose, kidney/adrenal, liver, small intestine, muscle, chondrocytes and ovary.

Copyright © 2004 John Wiley & Sons, Ltd. 

that represent the immune, metabolic/somatic, neuroendocrine and reproductive systems [9]. [Note: The cost of normalization and sequencing of liver and fat cDNA libraries was shared with Robin Morgan and Joan Burnside at the University of Delaware (UD), Delaware Biotechnology Institute (DBI) under a USDA–NRI/Animal Genome Program, Molecular Tools and Reagents Grant]. On 17 December 2001, the British Biotechnology and Biological Sciences Research Council (BBSRC) Chicken EST Consortium released an extensive database of almost 300 000 chicken ESTs (**http://www.chick.umist.ac.uk/index.html**). This large number of chicken ESTs was derived from major organs and cell types of Leghorn (egg-type) chickens, with a strong emphasis on early embryonic development [10]. The UD collection contains a large number of ESTs sequenced from unique cDNA libraries [i.e. various lymphoid tissues, abdominal fat, epiphyseal growth plate, pituitary, hypothalamus, pineal, oviduct (with egg in transit), and testes] of broiler (meat-type) chickens. Accordingly, these two EST collections are complementary, and the UD chicken EST collection has minimal overlap with the ESTs sequenced from 21 normalized libraries by the BBSRC consortium (Table 1).

All chicken ESTs (407 K) found in public databases (on 1 March 2003) were assembled by the CAP3 program [11] into 33 949 high-fidelity contigs (Figure 1) that could represent the number of bona fide genes expressed in the chicken [12]. This overall chicken EST assembly greatly enhanced gene identification and clustering of unigene sets from our cDNA libraries (Figure 1A). Before CAP3 assembly of all public chicken ESTs, about 52% of the 42 964 ESTs in the UD collection had a high (>200) BLASTX score, while 26% had a low BLASTX (<200) score and 22% were classified as unknown (Figure 1A). Gene identity based on BLASTX (or BLASTN) scores of the 12 537 UD contigs was improved by our CAP3 assembly

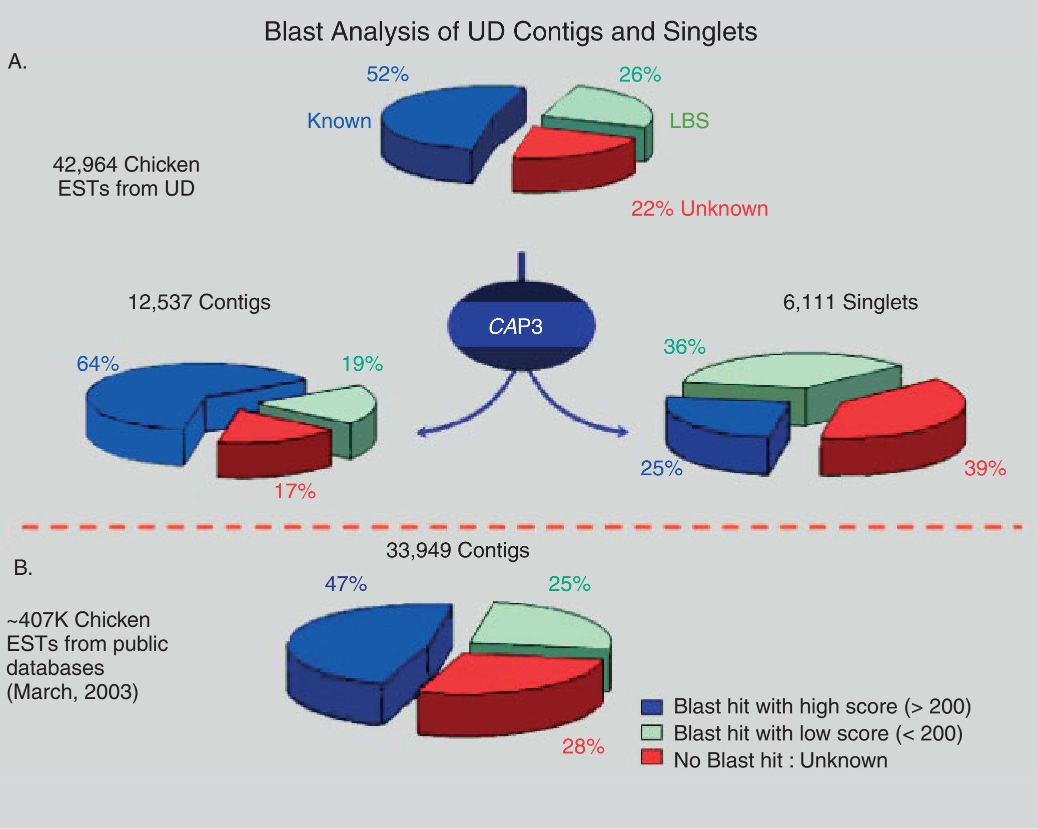

**Figure 1.** CAP3 assembly of (A) 42 964 ESTs in the UD collection and (B) all chicken ESTs (~407 K) in found in public databases [12]. The CAP3 program was used at a stringency of 40 bp overlap and 90% sequence identity

Copyright © 2004 John Wiley & Sons, Ltd. 

of all public chicken ESTs to 64% with high BLAST scores, 19% with low scores and 17% with no identification. Thus, the UD chicken EST collection contains 18 648 non-redundant sequences, with an overall redundancy rate of 4.6 ESTs/contig as compared to 8.3 ESTs/contig for the larger BBSRC collection. The UD CAP3 assemblies were used to construct a Chicken Gene Index with 33 949 contigs (high-fidelity *in silico* cDNAs) and 84 070 unclustered singlets [9]. Furthermore, our assembly of publicly available chicken ESTs is in good agreement with The Institute for Genome Research (TIGR) *Gallus gallus* gene index, GgGI, Version 5.0 of which was also built using the CAP3 program. The UD CAP3 assembly has allowed us to establish a non-redundant set of genes from liver, adipose tissue, breast (white fibres) and leg (red fibres) muscle/epiphyseal growth plate, pituitary gland/hypothalamus/pineal, reproductive tract (Figure 2) and lymphoid tissues.

Thus, the UD chicken EST collection is based on three major physiological systems (metabolic/somatic, neuroendocrine/reproductive and immune) [9]. Furthermore, the UD CAP3 database contains all information stemming from this assembly (i.e. the detailed alignment of contigs, EST sequences used to build contigs, BLASTN and BLASTX reports, and the PubMed links in GenBank). The UD chicken EST database (**http://www.chickest.udel.edu**) and our CAP3 assemblies (**http://udgenome.ags.udel.edu/cogburn/**) can be searched by nucleotide sequence or keyword. A portion of the UD EST collection (23 427 ESTs) was recently exploited for single nucleotide polymorphism (SNPs) discovery by another UD group [13] (**http://chicksnps.afs.udel.edu**).

### Development of chicken cDNA microarrays

A primer on the principles of microarray technology and its application to poultry genetics, breeding and biotechnology has recently been published [14]. Prior to this, there had been only a few papers published on gene expression profiling with chicken DNA microarrays [7,15–18]. Low-density arrays and differential mRNA display were used to examine the chicken's auditory system (i.e. the cochlea and brain) for auditory plasticity [15]. The first chicken lymphoid cDNA microarrays (1–3 K) were derived from about 5251 ESTs sequenced from an activated T cell cDNA library [7]. Two of

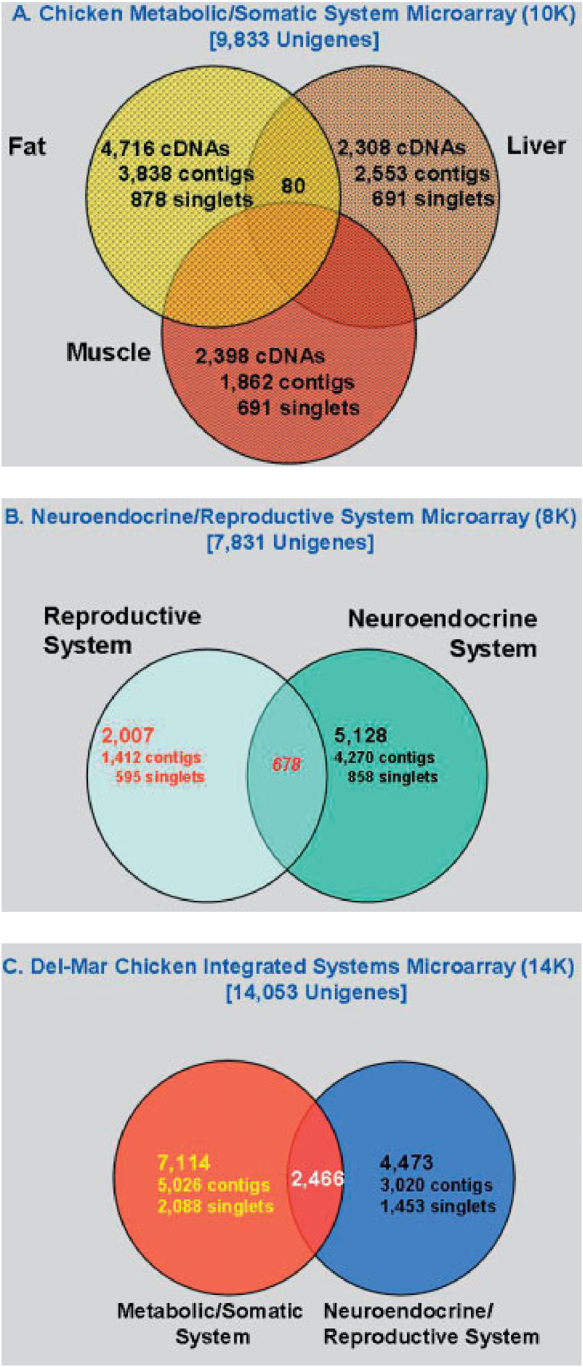

**Figure 2.** Venn diagrams of tissue-specific chicken unigene sets that were integrated into the Del-Mar Chicken Microarrays these initial microarray studies have given us the first look at global gene expression of the chicken innate immune system during normal development

Copyright © 2004 John Wiley & Sons, Ltd.

[17] or provoked responses [16]. An additional five papers were published on microarray analysis of chicken tissues in 2003; these interrogations of transcriptional units in the chicken genome involved tissue-specific DNA microarrays for liver [6], pineal [19], retina [20], intestine [21,22] and the bursa of Fabricius [23].

Under our USDA–IFAFS consortium project, we have developed and printed both tissue-specific and systems-wide chicken cDNA microarrays [6]. Our prototype liver-specific array (3.1 K unigenes) was printed on nylon membranes and used in several definitive studies [24–26]. The Chicken Metabolic/Somatic System (Figure 2A) and Neuroendocrine/Reproductive Systems (Figure 2B) microarrays were originally printed and used as independent arrays. Recently, we have combined both of these systems-wide gene sets into the Del-Mar 14K Chicken Integrated Systems Microarray (Figure 2C). This universal high-density microarray is currently being used for time-series transcriptional profiling across multiple tissues from divergently selected lines of broiler chickens [6].

An integrated immune system microarray (4 K) has been developed by Joan Burnside and Robin Morgan at UD, DBI from their collection of lymphoid ESTs. They currently use the lymph microarray for studies on the chicken's immune defence system. A chicken macrophage microarray (4 K) has been recently developed by another group at UD from several thousand chicken ESTs sequenced from activated-macrophage cDNA libraries [27] (**www.aviangenomics.udel.edu**). These ESTs have now brought the total number of chicken ESTs submitted to GenBank by the UD chicken genomics group to 47 853 (Table 1). Recently, a group from the Chicken Genome Consortium [Dave Burt, Roslin Institute; Joan Burnside, UD/DBI; Paul Neiman, Fred Hutchinson Cancer Research Center (FHCRC)] has developed a high-density (13 K) chicken cDNA array that mainly represents the high scoring BLASTX contigs from the BBSRC collection and a few thousand lymphoid clones. This generic 13 K chicken microarray is available in Europe from ARK-Genomics (**http://www.ark-genomics.org**) and in North America from the FHCRC (**genomics@fhcrc.org**). Currently, the FHCRC microarray appears to be the most widely used functional genomics platform available to the chicken genomics community. Furthermore, a Chicken GeneChip® is under development by Affymetrix (**http://www.affymetrix.com/index.affx**) and the Chicken Genome Consortium Microarray Committee (**http://www.chicken-genome.org/**) for release later this year.

### Modelling of gene networks and regulatory pathways

One of the most promising new developments in functional genomics is gene network modelling [28–31]. A strong external perturbation is applied and the transcriptional snapshots from time-series experiments are used to estimate the regulatory strengths of gene–gene interactions [28,29,32,33]. The perturbation method [34] is widely used in yeast and plants, where each gene in a pathway under study is perturbed, one gene at time. However, gene-by-gene perturbations are not practical in complex organisms like birds and mammals. We have used two strong metabolic perturbations — the embryo-to-hatching transition [25] and the fasting and re-feeding response [26] — to take time-series transcriptional snapshots of chicken liver. A dynamic Bayesian model for analysis of microarray data (BAM) and a spanning tree clustering method were developed for mapping 'functional' clusters of genes that respond to these metabolic perturbations [35].

### Global gene expression profiling in liver of the peri-hatch chick

We have examined global gene expression in the liver of embryos (e16, e18 and e20) and hatchling chicks (1, 3 and 9 days) during the very critical and vulnerable peri-hatch period [6]. A multidimensional projection of 32 clusters of functionally related genes expressed in the liver during the peri-hatch period is presented in Figure 3A. Two major and distinct patterns of gene expression were revealed from a total of 756 differentially expressed genes in this cluster tree. One group of 49 genes (red clusters) had higher levels of expression in embryos, whereas the opposing blue clusters had the opposite pattern, with higher expression after hatching (Figure 3B). Gene cluster analysis, using our spanning tree model, shows the interconnectivity of functional gene clusters involved in the metabolic switch from embryonic to terrestrial life in the peri-hatch chick. Several

Copyright © 2004 John Wiley & Sons, Ltd. 

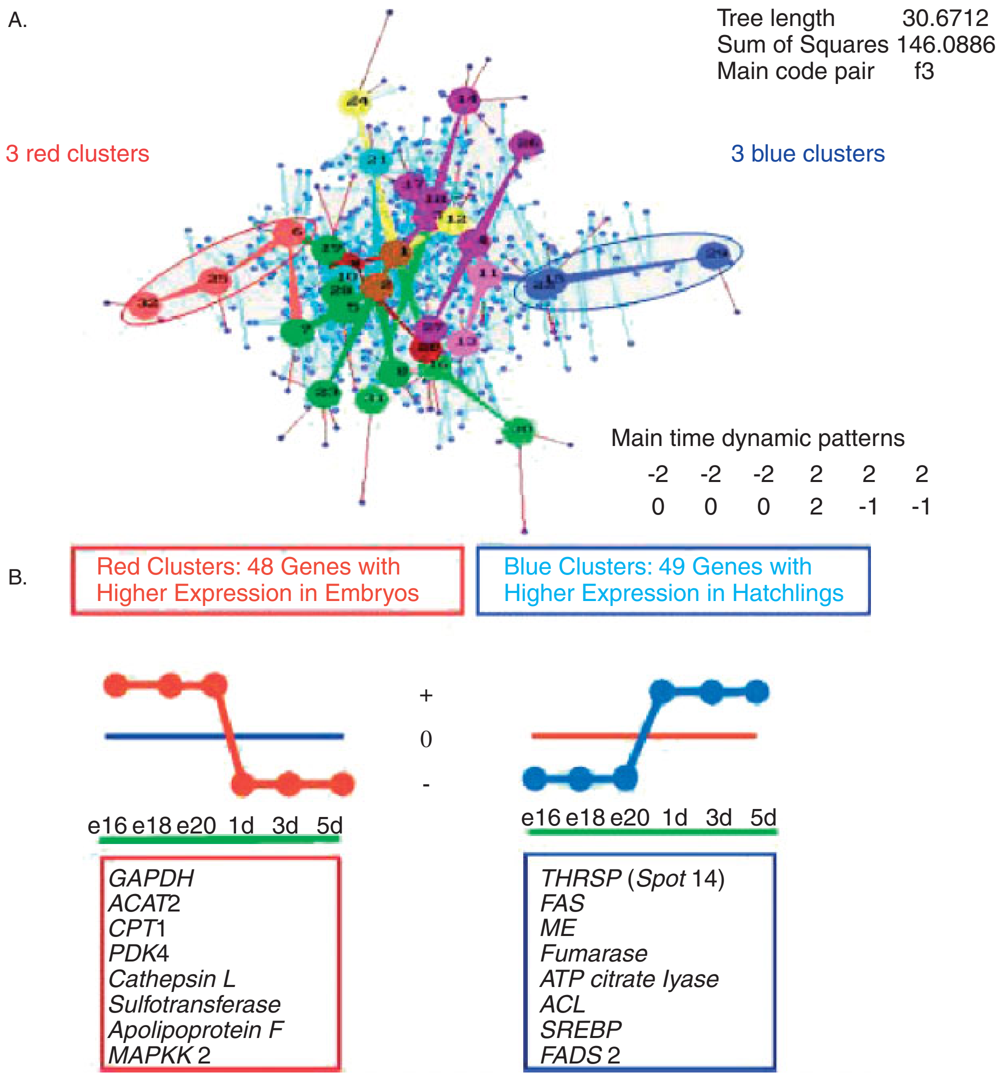

**Figure 3.** Cluster analysis of gene expression patterns in liver during the embryo-to-hatchling transition, using a liver-specific cDNA array (3.1 K). Opposing clusters of functionally related genes in this multidimensional tree also have opposing patterns of gene expression, e.g. the three red clusters contain genes whose expression is high during late embryonic development then fall after hatching; the opposing blue branch clusters contain the genes that are highly expressed after hatching. The inserts provide some examples of these functionally related genes. Liver samples for microarray analysis were collected at three embryonic (e) ages (e16,e18,e20) and at three ages after hatching [(1day) 1d, 3d and 9d]

enzymes, expressed at higher levels in embryos, are directly involved in fatty acid metabolism [acetyl coenzyme A acetyltransferase 2 (*ACAT2*), pyruvate dehydrogenase kinase 4 (*PDK4*) and carnitine palmitoyl-transferase 1 (*CPT1*)]. The opposing blue clusters contain a number of transcription factors and metabolic enzymes that are expressed at higher levels in the liver of the newly hatched chick; these genes are involved in lipogenesis and energy metabolism [thyroid hormone responsive *Spot 14* protein (*THRSP*), peroxisome proliferator-activated receptor-$\gamma$ (*PPAR*$\gamma$), CCAAT/enhancer-binding protein-$\alpha$ (*CEBP*$\alpha$), fatty acid synthase (*FAS*), malic enzyme (*ME*) and HMG CoA synthese (*HMG CS*)]. For example, *THRSP* (*Spot 14*) is a transcription factor which controls the expression of several enzymes in the lipogenic pathway (see Figure 4 in [6]). Furthermore, we have

Copyright © 2004 John Wiley & Sons, Ltd. 

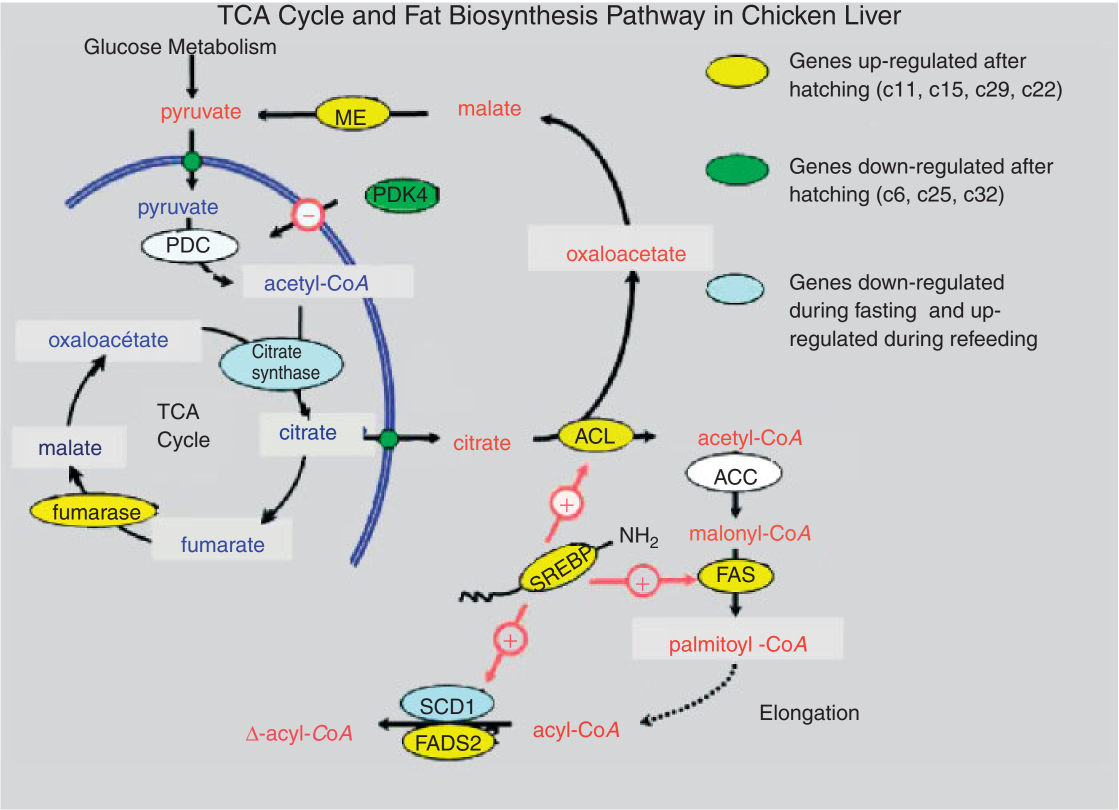

**Figure 4.** Transcriptional control of the TCA cycle and fat biosynthesis in chicken liver. This working model is based on functional clusters of genes identified from the analysis of two perturbation studies discovered an insertion/deletion polymorphism in chicken *THRSP* that is associated with abdominal fat traits [36]. Thus, time-series perturbation studies and gene cluster analysis provides a very powerful method for revealing the major topography of gene networks that control major metabolic pathways in chicken liver.

### Mapping of functional genes in metabolic pathways

Some of the metabolic enzymes and transcription factors identified by cluster analysis in the livers of the peri-hatch chick [6] or fasting and re-fed chickens [26] were integrated into a working model of transcriptional control of the TCA cycle and fat biosynthesis pathway (Figure 4). Several genes found in these functional clusters are directly involved in fatty acid metabolism [sterol response element binding protein (*SREBP*); ATP citrate lyase (*ACL*); *FAS; ME*; fatty acid desaturase 2 (*FADS2*)]. The metabolic genes found in these clusters agree with those known to regulate these pathways in mammals [37]. A number of genes (*Spot 14, ACL, FAS, FADS2)* are overexpressed after hatching and have the same expression pattern as *SREBP,* which regulates expression of lipogenic genes. The upregulation of fumarase in the TCA cycle and the production of acetyl CoA also contribute to increased lipogenesis. Further, pyruvate dehydrogenase kinase-4 (*PDK4*), which is upregulated in the liver of embryos, inhibits the activity of the pyruvate dehydrogenase complex (*PDC*) in the TCA cycle. Thus, downregulation of *PDK4* would contribute to increased lipogenesis by increasing the production of acetyl CoA in the mitochondria. Furthermore, *PDK4* is known to be upregulated by *PPARα*. *PPARα* promotes expression of genes involved in $\beta$-oxidation of fatty acids and overexpression of *PPARα* inhibits *SREBP* promoter activity in a dose-dependent manner [38]. In addition, *PPARα* levels are strongly upregulated in the liver of fasting chickens, which

Copyright © 2004 John Wiley & Sons, Ltd. 

reflects an increase in catabolism of stored fat. In contrast, *PPARγ* appears to support lipogenesis, since the hepatic expression of *PPARγ* is dramatically increased after hatching. Overexpression of genes in the lipogenic pathway and inhibition of the lipolytic pathway could be related to the nutritional transition between embryonic and hatchling metabolic states. Lipogenesis in the chick liver is very low during the embryonic period and the first few days after hatching [39,40]. In chickens, lipogenesis, although likely to be controlled by the same genes as in mammals, takes place primarily in the liver, whereas adipocytes serve for the release and storage of triglycerides. The balancing and partitioning of nutrients between metabolic tissues could be controlled in a different way in chickens and mammals. Mapping of transcriptional networks requires high-throughput analysis of microarray scans, clustering of co-regulated genes and computational analysis for the presence of functional motifs (i.e. *cis*-regulatory elements and transcription factor binding sites) that exert control over major metabolic pathways [41]. The assembly of the chicken genome sequence in the near future will certainly enhance efforts to understand transcriptional regulation of major gene networks.

### Conclusions

The current bonanza of genomic resources (460 K ESTs, several high-density microarrays and a complete genome sequence) for the chicken should soon shift the domestic chicken to the forefront of developmental biology and functional genomics research. We have constructed and normalized five tissue-specific chicken cDNA libraries and completed high-throughput sequencing of 30 609 ESTs. Chicken unigene sets were identified by CAP3 clustering for development of tissue-specific (liver) and systems-wide (metabolic/somatic and neuroendocrine) chicken DNA microarrays. Using gene clustering and computational analyses of time-series transcriptional profiles, we have identified a number of polymorphic functional genes in key metabolic pathways that could control important phenotypes in chickens.

### Acknowledgements

This work was supported by grants from the USDA–IFAFS (Award No. 00-52100-9614) Animal Genome Program and the Maryland Agricultural Experiment Station (TEP).

### References

1. Price EO. 2002. *Animal Domestication and Behavior*. CABI: Wallingford, UK.
2. Dohner JV. 2001. *The Encyclopedia of Historic and Endangered Livestock and Poultry Breeds*. Yale University Press: New Haven, CT.
3. Warren W. 2004. Plenary Lecture: Analysis of the chicken genome. Proceedings of Plant and Animal Genome XII Conference, San Diego, CA; p. 30. **http://www.intl-pag.org/12/12-session1.html**
4. Burt D, Pourquie O. 2003. Chicken genome: science nuggets to come soon. *Science* **300**: 1669.
5. Brown WRA, Hubbard SJ, Wilson SA. 2003. The chicken as a model for large-scale analysis of vertebrate gene function. *Nature Rev Genet* **4**: 87–98.
6. Cogburn LA, Wang X, Carre W, *et al.* 2003. Systems-wide chicken DNA microarrays, gene expression profiling and discovery of functional genes. *Poult Sci* **82**: 939–951.
7. Tirunagaru VG, Sofer L, Cui J, *et al.* 2000. An expressed sequence tag database of T cell-enriched activated splenocytes: sequence analysis of 5251 clones. *Genomics* **66**: 144–151.
8. Abdrakhmanov I, Lodygin D, Geroth P, *et al.* 2000. A large database of chicken bursal ESTs as a resource for the analysis of vertebrate gene function. *Genome Res* **10**: 2062–2069.
9. Cogburn LA, Carre W, Wang X, *et al.* 2004. Functional genomics resource: sequencing and annotation of 42 272 ESTs from single and multiple tissue cDNA libraries and CAP3 assembly of a chicken gene index (submitted).
10. Boardman PE, Sanz-Ezquerro J, Overton IM, *et al.* 2002. A comprehensive collection of chicken cDNAs. *Current Biol* **12**: 1965–1969.
11. Huang X, Madan A. 1999. CAP3: A DNA sequence assembly program. *Genome Res* **9**: 868–877.
12. Carre W, Niu Y, Gao GR, *et al.* 2003. The chicken gene index: CAP3 sequence assembly and applications for functional genomics. Proceedings of the Plant and Animal Genome XI Conference, San Diego, CA. **http://www.intl-pag.org/11/abstracts/P01_P61_XI.html**.
13. Kim H, Schmidt CJ, Decker KS, *et al.* 2003. A double-screening method to identify reliable candidate non-synonymous SNPs from chicken EST data. *Anim Genet* **34**: 249–254.
14. Cogburn LA, Morgan R, Burnside J. 2003. Expressed sequence tags, DNA chip technology and gene expression profiling. In *Poultry Genetics, Breeding and Biotechnology*, Muir WM, Aggrey SE (eds). CABI: Wallingford; 629–646.
15. Lomax MI, Huang L, Cho Y, *et al.* 2000. Differential display and gene arrays to examine auditory plasticity. *Hear Res* **147**: 293–302.
16. Morgan RW, Sofer L, Anderson AS, *et al.* 2001. Induction of host gene expression following infection of chicken embryo fibroblasts with oncogenic Marek's disease virus. *J Virol* **75**: 533–539.
17. Neiman PE, Ruddell A, Jasoni C, *et al.* 2001. Analysis of gene expression during myc oncogene-induced lymphomagenesis in the bursa of Fabricius. *Proc Natl Acad Sci USA* **98**: 6378–6383.
18. Liu HC, Cheng HH, Tirunagaru VG, *et al.* 2001. A strategy to identify positional candidate genes conferring Marek's

Copyright © 2004 John Wiley & Sons, Ltd. 

disease resistance by integrating DNA microarrays and genetic mapping. *Anim Genet* **32**: 351–359.
19. Bailey MJ, Beremand PD, Hammer R, *et al.* 2003. Transcriptional profiling of the chick pineal gland, a photoreceptive circadian oscillator and pacemaker. *Mol Endocrinol* **17**: 2084–2095.
20. Hackam AS, Bradford RL, Bakhru RN, *et al.* 2003. Gene discovery in the embryonic chick retina. *Mol Vis* **9**: 262–276.
21. van Hemert S, Ebbelaar BH, Smits MA, *et al.* 2004. Generation of EST and microarray resources for functional genomics studies on chicken intestinal health. *Anim Biotechnol* **14**: 133–143.
22. Min W, Lillehoj HS, Kim S, *et al.* 2003. Profiling local gene expression changes associated with *Eimeria maxima* and *Eimeria accerulina* using cDNA microarray. *Appl Microbiol Biotechnol* **62**: 392–399.
23. Neiman PE, Grbic JJ, Polony TS, *et al.* 2003. Functional genomic analysis reveals distinct neoplastic phenotypes associated with *c-myb* mutation in the bursa of Fabricius. *Oncogene* **22**: 1073–1086.
24. Wang X, Carre W, Rejto L, *et al.* 2002. Global gene expression profiling in liver of thyroid manipulated and/or growth hormone (GH) injected broiler chickens. *Poult Sci* **81**(suppl 1): 63.
25. Glass B, Wang X, Carre W, *et al.* 2002. DNA microarray analysis of liver genes during the metabolic jump from chorioallantoic to pulmonary respiration. *Poult Sci* **81**: 31.
26. Duclos MJ, Wang X, Carre W, *et al.* 2004. Nutritional regulation of global gene expression in chicken liver during fasting and re-feeding. Proceedings of the Plant and Animal Genome XII Conference, San Diego, CA. **http://www.intl-pag.org/12/abstracts/P7b_PAG12_889.html**
27. Keeler CL, Emara MG, Bliss T. 2003. Construction and characterization of avian macrophage EST libraries. Proceedings of the Plant and Animal Genome XI Conference, San Diego, CA. **http://www.intl-pag.org/11/abstracts/P01_P62_XI.html**
28. de la Fuente A, Brazhnik P, Mendes P. 2002. Linking the genes: inferring quantitative gene networks from microarray data. *Trends Genet* **18**: 395–398.
29. Brazhnik P, Fuente A, Mendes P. 2002. Gene networks: how to put the function in genomics. *Trends Biotechnol* **20**: 467–472.
30. Klamt S, Stelling J. 2003. Two approaches for metabolic pathway analysis? *Trends Biotechnol* **21**: 64–69.
31. Papin JA, Price ND, Wiback SJ, *et al.* 2003. Metabolic pathways in the post-genome era. *Trends Biochem Sci* **28**: 250–258.
32. de la Fuente A, Snoep JL, Westerhoff HV, *et al.* 2002. Metabolic control in integrated biochemical systems. *Eur J Biochem* **269**: 4399–4408.
33. Stark J, Callard R, Hubank M. 2003. From the top down: towards a predictive biology of signalling networks. *Trends Biotechnol* **21**: 290–293.
34. Wagner A. 2001. How to reconstruct a large genetic network from $n$ gene perturbations in fewer than $n2$ easy steps. *Bioinformatics* **17**: 1183–1197.
35. Rejto L, Tusnady G. 2002. Assessing gene expression measurements. In *Computing Science and Statistics: Geoscience and Remote Sensing*, *Proceedings of the 34th Symposium Interface*, Wegman E, Braverman A (eds). Interface Foundation of North America: Fairfax Station, VA; pp. 42–56.
36. Wang X, Carre W, Zhou H, *et al.* 2004. Duplicated *Spot 14* genes in the chicken: characterization and identification of polymorphisms associated with abdominal fat traits. *Gene* (in press).
37. Unger RH. 2003. Lipid overload and overflow: metabolic trauma and the metabolic syndrome. *Trends Endocrinol Metab* **14**: 398–403.
38. Duplus E, Forest C. 2002. Is there a single mechanism for fatty acid regulation of gene transcription? *Biochem Pharm* **64**: 893–901.
39. Morris SM, Winberry LK, Fisch JE, *et al.* 1984. Developmental and nutritional regulation of the messenger RNAs for fatty acid synthase, malic enzyme and albumin in the livers of embryonic and newly hatched chicks. *Mol Cell Biochem* **64**: 63–68.
40. Goodridge AG, Fantozzi DA, Klautky SA, *et al.* 1991. Nutritional and hormonal regulation of genes for lipogenic enzymes. *Proc Nutr Soc* **50**: 115–122.
41. Banerjee N, Zhang MQ. 2002. Functional genomics as applied to mapping transcription regulatory networks. *Curr Opin Microbiol* **5**: 313–317.

Copyright © 2004 John Wiley & Sons, Ltd. 
